# Lateral Antimicrobial Resistance Genetic Transfer is active in the open environment

**DOI:** 10.1038/s41598-017-00600-2

**Published:** 2017-03-31

**Authors:** Luciana S. Chamosa, Verónica E. Álvarez, Maximiliano Nardelli, María Paula Quiroga, Marcelo H. Cassini, Daniela Centrón

**Affiliations:** 1 Instituto de Microbiología y Parasitología Médica, Facultad de Medicina, Universidad de Buenos Aires-Consejo Nacional de Investigaciones, Científicas y Tecnológicas (IMPaM, UBA-CONICET), Ciudad Autónoma de Buenos Aires, Argentina; 2 Grupo GEMA, Departamento de Ciencias Básicas, Universidad Nacional de Luján, Luján, Buenos Aires, Argentina; 3 Laboratorio de Biología del Comportamiento, IBYME, Ciudad Autónoma de Buenos Aires, Argentina

## Abstract

Historically, the environment has been viewed as a passive deposit of antimicrobial resistance mechanisms, where bacteria show biological cost for maintenance of these genes. Thus, in the absence of antimicrobial pressure, it is expected that they disappear from environmental bacterial communities. To test this scenario, we studied native IntI1 functionality of 11 class 1 integron-positive environmental strains of distant genera collected in cold and subtropical forests of Argentina. We found natural competence and successful site-specific insertion with no significant fitness cost of both *aadB* and *bla*_VIM-2_ antimicrobial resistance gene cassettes, in a model system without antibiotic pressure. A bidirectional flow of antimicrobial resistance gene cassettes between natural and nosocomial habitats is proposed, which implies an active role of the open environment as a reservoir, recipient and source of antimicrobial resistance mechanisms, outlining an environmental threat where novel concepts of rational use of antibiotics are extremely urgent and mandatory.

## Introduction

In April 2014, the World Health Organization published the first global report on antibiotic resistance, revealing that “this serious threat is no longer a prediction for the future, but it is happening in every region of the world and has the potential to affect anyone, of any age, in any country” (http://www.who.int/mediacentre/news/releases/2014/amr-report/en/). Although diverse strategies to understand and combat this global concern have been conceived and implemented^1–5^, the extent of the processes involved in the constant emergence of new mechanisms of resistance and its consequent dissemination worldwide is not yet clear.

Current trends in the rational use of antibiotics envisage multidrug resistance adaptation as a problem that involves, not only the nosocomial settings and highly anthropomorphized habitats, but also natural and semi-natural environments as potential reservoirs of resistance genes^6–11^. In this context, it is imperative that the scientific community not only investigates the underlying mechanisms for the distribution of antimicrobial resistance genes in the clinics but also the ecological dynamics of the dispersal of antibiotic resistance under the framework of the Lateral Genetic Transfer pathways, in order to build a comprehensive theory that integrates proximal and ultimate causes of this global threat to human health.

Among the diverse processes involved in the Lateral Antimicrobial Resistance Genetic Transfer (LARGT), the class 1 integron is the most efficient mechanism involved in the expression, recruitment, maintenance and spreading of resistance genes among Gram-negative clinical isolates^12–17^. The *intI1* gene codes a tyrosine recombinase which catalyses site-specific recombination reactions of different traits from the floating genome, independently of the phylogenetic relationships between donors and recipients^18, 19^, and without interrupting genes of the host^19, 20^. The gene cassettes are the mobile part of the two component integron/cassettes system, which can be inserted or excised from the integron’s variable region by IntI by site-specific recombination, implying that antimicrobial resistance gene cassettes (ARGCs) need functional integrases from integrons to invade novel target sites. In the hospital niche, the detection of the *intI1* gene in clinical strains is considered a genetic marker for multidrug resistance^16^, and some clones can be rapidly spread and maintained in outbreaks due, in part, to its acquisition^21^. The occurrence of class 1 integrons in the open environment is considered a common phenomenon^7, 14, 22, 23^, including habitats with low level of urbanization^22^, such as Tierra del Fuego Island, one of the most pristine environments of the world^22^. The epidemiological factors﻿ involved in their rapid dissemination associated to antibiotic resistance around the world are still not clear. Based on mathematical modelling, it has been recently proposed that functional class 1 integron integrases are expected to decline in frequency when a bacterial population is well adapted to a stable environment^24^. However, the functionality of class 1 integron integrases has been restricted to clinical isolates, and the recombinational properties of environmental class 1 integron integrases, with the eventual dissemination to the clinic, remains largely unknown.

Class 1 integrons are an appropriate model to investigate the ability of environmental bacterial strains to acquire antibiotic resistance gene determinants, for several genetic, architectural and epidemiological attributes. The two components integron/cassettes are closely related in the same mobile genetic platform, where the ARGCs (the antimicrobial resistance determinants in this model) are “﻿captured” by IntI1, inserted into the variable region and selected under antimicrobial pressure. Based on this feature, this complete genetic platform has been disseminating globally since the beginning of the antibiotic era till now  in clinical samples, with the ability to renew itself over time by exchanging ARGCs already described and/by putative new ones. Since they have been found in several habitats^7, 8, 14, 22, 23^, including hospitals and open environment, their provenance can be inferred by the type of allele of the *intI1* gene, “environmental” or “clinical”, which has been used as a biomarker to infer the directionality of the strains^22, 23, 25^. These features convert class 1 integrons as a suitable biological model to study the flow and the exchange of antimicrobial resistance determinants from the open environment to the clinic, and vice versa.

**Figure 1:**
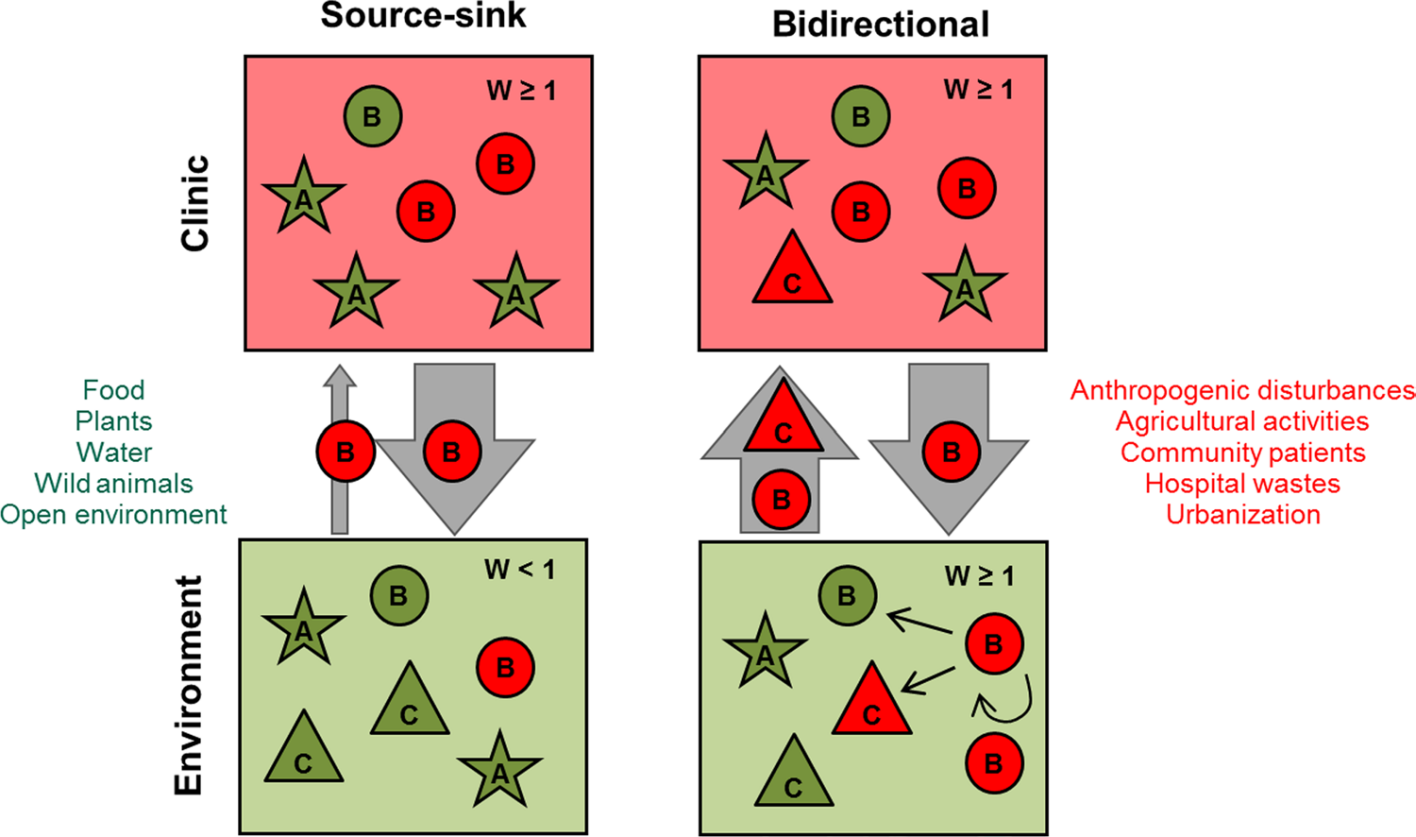
Two model systems of dissemination of antimicrobial resistance gene cassettes. In the source-sink model system there is a flow (grey arrow) from the clinic (source) to the open environment (sink). However, few events of recombination linked to antimicrobial resistance can be maintained in the environment (W < 1). In the bidirectional model system, clinical and environmental bacteria disseminate in both habitats. These bacteria from different niches can exchange and maintain antimicrobial resistance gene cassettes (ARGCs) (W ≥ 1) acquiring new phenotypes or not (Supplementary Table S1). Both the open environment and hospitals act as a reservoir and source of ARGCs able to deal with antibiotic pressure, while integrons modulate this exchange in part; arrows suggest possible ways of bacterial dissemination between settings. With the bidirectional model, the results are (i) a greater presence of ARGCs in the environment and in the clinical habitat, and (ii) the possibility that strains with new ARGCs penetrate in the hospital environment. Different shapes and letters represent distinct bacterial species or strains that can inhabit diverse micro-habitats. Red and green represent strains with or without ARGCs, respectively. Arrow width represents the amount of flux of resistant strains between habitats.

Most present literature conceives the environment as a passive drain of antimicrobial resistance mechanisms, which will disappear over time in the absence of a flux of bacteria from nosocomial environments, and of antimicrobial pressure^26, 27^. This dynamic emulates a source-sink system, as it was extensively described in ecological literature to describe meta-population dynamics^7, 28, 29^ (Fig. 1). The aim of this work was to test the following assumptions of the source-sink model: (i) in environments without antimicrobial pressure, fitness of bacterial strains containing resistance genes should be lower than strains without these genes; (ii) LARGT in the environment is a rare phenomenon, at least it has not been examined experimentally with non-clinical strains; and (iii) without the constant flow from the clinics (source), the antimicrobial resistance genes should disappear from the environment (sink).

We tested these assumptions by following five steps: (1) we selected strains collected in natural areas far from nosocomial sites with complete *intI1* genes; (2) we evaluated these environmental strains for the functionality of the native IntI1 in a model system of recombination at wild-type levels of integrase expression, without performing antibiotic selection; (3) we evaluated whether these environmental strains were able to acquire ARGCs without human intervention by using natural transformation; (4) we determined the sequence of molecular mechanisms involved in the transfer, and acquisition of ARGCs; and (5) we investigated whether there was biological cost for the acquisition of ARGCs. Working under the biological model system of class 1 integrons previously stated, we have identified *intI1*-positive environmental strains isolated from diverse regions of Argentina able to insert ARGCs in their native *attI1*, even at higher frequencies than already described for class 1 integron integrases found in clinical samples. We have also found that the biological cost of both ARGCs tested in this study was neutral in the host *intI1*-positive environmental strains, pointing out a more dynamic bidirectional model of clinical-environmental exchange of antimicrobial resistance determinants, which is proposed in the present study.

## Results and Discussion

### Native IntI1s are able to acquire ARGCs

**Figure 2:**
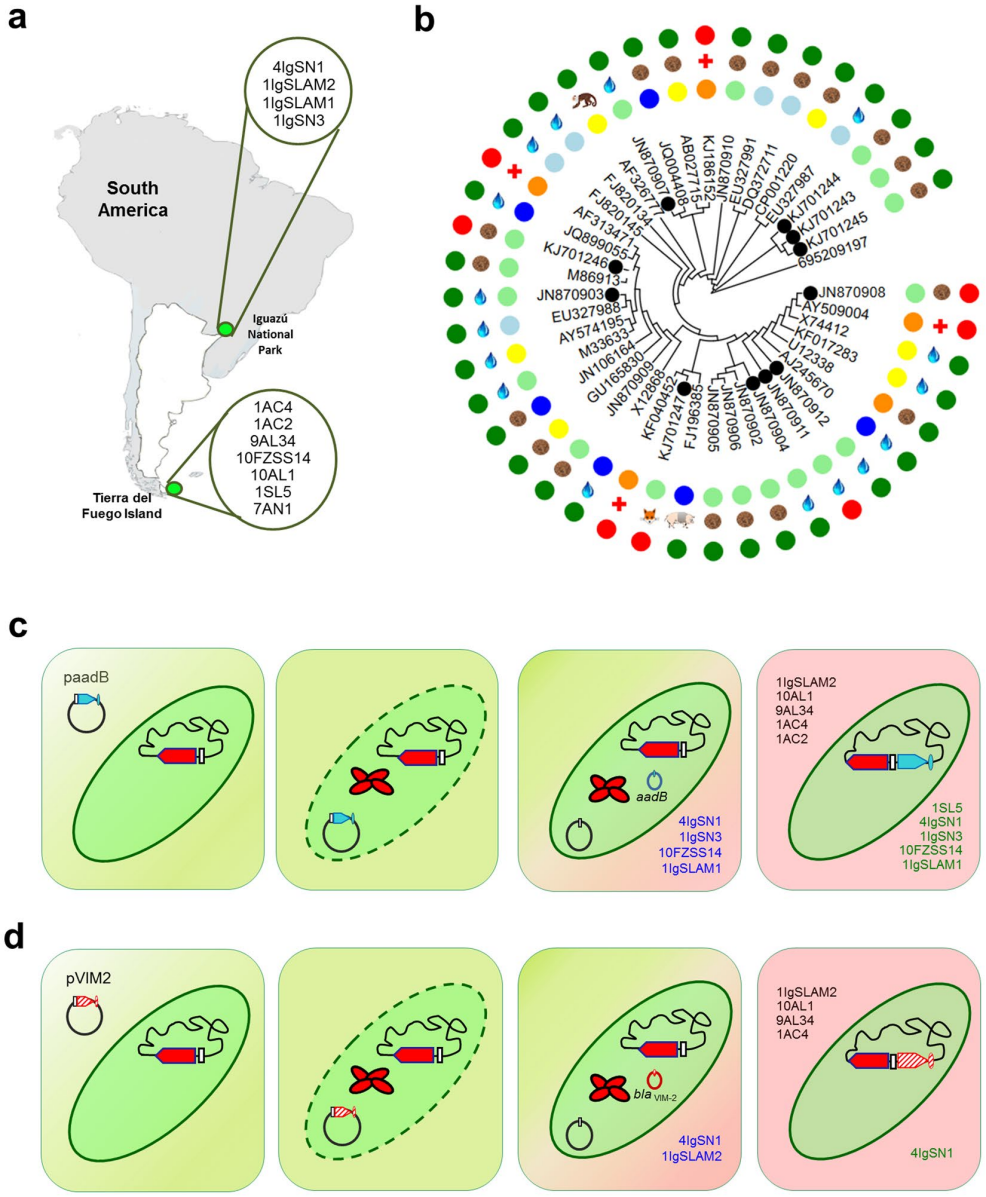
Model system of environmental *intI1*-positive strains used for ARGC acquisition. (**a**) Origin of environmental strains. *Escherichia coli* 4IgSN1, *Enterobacter* sp. 1IgSLAM2, *Acinetobacter* sp. 1IgSLAM1 and *Acinetobacter* sp. 1IgSN3 were isolated in Iguazú National Park in Misiones Province, whereas, *Pseudomonas* sp. 1SL5, *Enterobacter* sp. 10AL1, *Aranicola* sp. 9AL34, *Pseudomonas* sp. 7AN1, *Aeromonas media* 1AC2, *Vibrio* sp. 1AC4 and *Pantoea dispersa* 10FZSS14 were obtained from Tierra del Fuego Island, Argentina. South America map was modified from public domain artwork (https://openclipart.org/detail/181050/argentina-location). (**b**) Phylogenetic tree of environmental *intI1* alleles. A total of 36 alleles of the *intI1* gene were identified from a BLASTn query using AF313471 as a reference (April 2015). Black dotted circles correspond to environmental strains used in this study. The inner lane corresponds to the provenance of the *intI1*-positive strain: blue for Europe, yellow for Asia, light blue for Oceania, light green for America and orange for pandemic dissemination. The medium lane corresponds to the type of sample: water drop for water sample, brown circle for soil, monkey for monkey faeces sample, pig for pig faeces sample and fox for fox faeces sample. The outer lane corresponds to the clinical (red circle) or environmental (green circle) source of the strain. (**c** and **d**) Steps of ARGC acquisition without antibiotic selection. Environmental isolates were subjected to artificial and natural transformation with plasmids paadB (**c**), carrying the *aadB* gene cassette, and pVIM2 (**d**), carrying the *bla*_VIM-2_ gene cassette. After transformation, functional native IntI1 excised the ARGC and inserted it in the native *attI1* site. Plasmid maintenance during 40 generations was observed in environmental strains that are in blue letters. Environmental strains able to perform ARGCs acquisition by both natural and artificial transformation are in green letters, while isolates that only insert the ARGC as a result of an artificial transformation are in black letters.

In order to simulate non-clinical conditions, environmental *intI1* positive-strains were used in a model of ARGCs recombination without antimicrobial selection (see Methods). This methodological approach differed from previous similar studies which were performed with *intI1* “clinical” alleles^23^ under antibiotic pressure, usually with overexpressed integrases in *E*. *coli* ^30–39^. Eleven environmental strains belonging to 8 genera of γ-*Proteobacteria* harbouring the complete *intI1* gene, isolated from different substrates, including soil, river water and fox faeces, were selected for recombination assays (Supplementary Table S1, Fig. 2). Five out of the 11 environmental strains were found in natural habitats far from urban areas^22^ with low levels of urbanization (Supplementary Table S1, Fig. 2a and b, see Methods and Study Area description in ref. 22). Seven out of the 11 *intI1* genes were novel “environmental” alleles, which had not been previously studied in recombination assays (Table 1). When we searched environmental *intI1* genes in GenBank (April 2015), we identified 36 “﻿environmental” *intI1* alleles, including 29 species belonging to 16 genera from γ and β Proteobacteria and Actinobacteria isolates (Fig. 2). This wide dissemination between habitats and genera around the world shows the global distribution of class 1 integrons in nature.

**Table 1:** Type of *intI1* allele (“clinical” or “environmental”), Pc variants, *intI1* expression at different conditions and insertion frequencies of the ARGC (*aadB* or *bla*_VIM-2_ gene cassette) in the native *attI1* sites of the environmental strains.

Isolate	Type of *intI1* allele	Pc variant	IntI1 name	Insertion frequencies (%)		Condition			
				*aadB* cassette	*bla*_VIM-2_ cassette	24-hours culture without treatment	24 hours post chemical transformation with paadB	24 hours post chemical transformation without any plasmid	24 hours post natural transformation assay
*Pseudomonas* sp. 1SL5	“Clinical”	PcS	IntI1__R32_N39_	7.88	−	−	+	−	−
*Acinetobacter* sp. 1IgSLAM1	“Environmental”	PcW	IntI1__R32_H39_	0.02	−	−	+	+	+
*Enterobacter* sp. 10AL1	“Environmental”	PcW	IntI1__R32_H39_	0.06	0.01	−	+	−	ND
*Aranicola* sp. 9AL34	“Environmental”	PcW	IntI1__R32_H39_	0.10	0.02	−	+	−	ND
*Pseudomonas* sp. 7AN1	“Environmental”	PcS	IntI1__R32_N39_	−	−	−	−	−	ND
*Aeromonas media* 1AC2	“Clinical”	PcH2	IntI1__R32_N39_	0.10	−	−	−	−	ND
*Vibrio* sp. 1AC4	“Environmental”	PcW	IntI1__R32_H39_	0.12	0.0003	−	−	−	ND
*Pantoea dispersa* 10FZSS14	“Clinical”	PcH1	IntI1__R32_H39_	1.01	−	+	+	+	+
*Enterobacter* sp. 1IgSLAM2	“Environmental”	PcW	IntI1__R32_H39_	0.004	0.003	−	−	−	ND
*Acinetobacter* sp. 1IgSN3	“Environmental”	PcW	IntI1__R32_H39_	0.09	−	−	+	+	+
*Escherichia coli* 4IgSN1	“Clinical”	PcW	IntI1__R32_H39_	0.88	0.0002	−	+	+	−
*Escherichia coli* TOP10 pMI1-1 with selection^a^	“Clinical”	PcW	IntI1__R32_H39_	9.71	5.85	+	+	+	ND
*Escherichia coli* TOP10 pMI1-1 without selection^b^	“Clinical”	PcW	IntI1__R32_H39_	0.09	0.07	−	−	−	ND

The 11 *intI1*-positive environmental strains were artificially transformed with plasmid paadB or pVIM2 in order to estimate the insertion frequencies of *aadB* and *bla*_VIM-2_ gene cassettes, respectively. *E*. *coli* TOP10 pMI1-1 was used to compare results. − indicates a negative result. Insertion frequencies are the mean value of four independent assays.

^a^Transformants cells were positively selected by the addition of ampicillin (100 µg/ml) and kanamicin (25 µg/ml).

^b^Transformants cells were not positively selected by the addition of antibiotics. In ^a^ and ^b^ *intI1* expression was induced by the supplement of IPTG (2 mM).

The *intI1* transcription was evaluated by RT-PCR in four conditions. + and − indicate positive and negative RT-PCR amplifications, respectively. Each assay was repeated three times. ND: not determined.

In three independent experiments, we introduced the paadB (harbouring the *aadB* gene cassette that confers resistance to gentamicin), or the pVIM2 (harbouring the *bla*_VIM-2_ gene cassette that confers resistance to carbapenems) plasmids by artificial transformation^40^, in each environmental strain (Fig. 2c, see Methods). Success of the corresponding ARGC insertion in the native *attI1* site after the artificial transformation was determined by PCR and sequencing the amplicon (Fig. 2c, Supplementary Table S2, see Methods). Positive results for insertion of the *aadB* gene cassette in native *attI1* sites were found in all environmental strains except *Pseudomonas* sp. 7AN1 (Table 1). The *bla*_VIM-2_ gene cassette was inserted into *Enterobacteriaceae* strains and in *Vibrio* sp. 1AC4 at the respective native *attI1* site (Fig. 2c, Table 1). The sequence analysis of the amplicons generated for assessing insertion of the ARGC (Supplementary Table S2), revealed that the recombination was site-specific in the native *attI1* site of the class 1 integrons.

### High frequencies of ARGC acquisition by native IntI1s

A wide range of insertion frequencies for both ARGCs was found by qPCR analyses (Table 1). Both *Pseudomonas* sp. 1SL5 and *Pantoea dispersa* 10FZSS14, each containing a common “clinical” allele of *intI1* (JN870908 and KJ701247, respectively), showed the highest frequencies for the insertion of the *aadB* gene cassette. The insertion frequency of the *aadB* gene cassette in the native site *attI1* of *Pseudomonas* sp. 1SL5 was 88 times higher than the control of the *E*. *coli* TOP10 co-transformed with two plasmids (pMI1-1 as donor of clinical *intI1* with the respective *attI1* site, and paadB as donor of the *aadB* gene cassette) without antimicrobial selection, and 1750 times higher than *Enterobacter* sp. 1IgSLAM2 isolate (Table 1). Interestingly, the frequency of insertion of the *aadB* gene cassette in the traditional control *E*. *coli* TOP10::pMI1-1::paadB, was lower than the frequencies observed for other environmental strains in our model system without antimicrobial selection (Table 1). In the case of the *bla*_VIM-2_ gene cassette, all 5 positive environmental strains harboured at least 100-times lower frequencies than that shown by *Pseudomonas* sp. 1SL5 for insertion of the *aadB* gene cassette. The most active environmental isolate in the acquisition of *bla*_VIM-2_ gene cassette was *Aranicola* sp. 9AL34, which possesses the environmental allele with 9 mutations compared to “clinical” *intI1* alleles^22^. We also performed an experiment in a model system with antibiotic pressure, in which we selected with kanamycin and ampicillin for the maintenance of both donor plasmids (pMI1-1 and paadB or pMI1-1 and pVIM2), as in the previous experiment with *E*. *coli* laboratory strains done in earlier recombination studies that analyzed IntI1 activities from several laboratories^31–35^. In this case, as expected by the antimicrobial selection of plasmids, an increase of 10- to 100-times of the insertion frequency was observed for both ARGCs (Table 1), as evidenced in previous studies^31–35^. In order to understand which features allow a highest activity of some *intI1*-positive environmental isolates in non selective experiments, several genetic and ecological properties of class 1 integrons have been analyzed. On one hand, it is likely that in absence of selective pressure on the inserted ARGC as in the previous experiments with *intI1*-positive environmental strains from this work, IntI1 is able to excise the respective ARGC, which probably explains at least in part the differences in integration rates between selective and non selective experiments for *E. coli* TOP10 (Table 1). Amongst the 11 *intI1*-positive environmental isolates, 4 were “clinical” alleles already described (*Pantoea dispersa* 10FZSS14, *Pseudomonas* sp. 1SL5, *E*. *coli* 4IgSN1 and *Aeromonas media* 1AC2 were identical to AN: DQ247972, AY463797, AM412236 and CP000650, respectively) and 7 were novel “environmental” *intI1* alleles (Table 1). In turn, the *intI1* genes from this study harbored 4 variants of Pc promoter^41, 42^ (Table 1). No association was found between “environmental” or “clinical” alleles of *intI1* and the Pc promoter variant. We have also analyzed if there were mutations related to IntI1 catalytic sites^43–45^. We found that 9 out of the 11 *intI1* genes had a total of 20 non-synonymous mutations throughout the gene compared to “clinical” allele (U49101). Although *Acinetobacter* sp. 1IgSLAM1 and *Acinetobacter *sp. 1IgSN3 had the same residue substitution in the ALER motif (Data not shown), which is an important region for IntI1 functionality, their insertion frequencies were not the lowest as shown in Table 1. No common variations to all environmental IntI1 have been identified in our study sample. On the other hand, the different frequencies observed in environmental strains cannot be neither fully explained by the process of recombination mediated by IntI1, or by the efficiency of transformation itself. The presence of more than one *intI1* gene in a cell, or the location of *intI1* in a high copy number plasmid, might be also involved in the different levels of recombination. These features were not investigated because they were outside the scope of the present work. Only when the *intI1* expression was investigated 24 hours post chemical transformation with paadB (Table 1), we identified that low values of *aadB* insertion at the native *attI1*, were in agreement with no detection of *intI1* expression. However, this cannot be taken as an explanation of the observed differences in recombination frequencies, but rather a consequence of the event. Host factors such as IHF, FIS, H-NS or LexA^46^, or even undetermined ones might be also involved in the variations observed in insertion frequencies. It is likely that the combination of many of these processes in each environmental strain is related to the variations observed in the frequencies of ARGC insertion at the native *attI1*. These experimental data constitute the first indication of the multifactorial relevance that functional environmental class 1 integron integrases would have, not only in the open environment but also in their passage to the clinical habitat.

As a whole, these findings show that environmental strains with several native “clinical” or “environmental” alleles of the *intI1* gene, from low or high level of urbanization, from different habitats (soil, sediment or microbiota of wild animals), with different strengths of Pc promoter, and harbouring novel *attI1* sites (Supplementary Figure S1), were active for the acquisition of ARGCs without antimicrobial pressure. Furthermore, some of the environmental strains showed higher frequencies than those observed in the traditional model system with antibiotic selection.

### Natural Competence also involved in ARGC capture

With the aim of identifying whether bacterial isolates from the open environment were able to acquire exogenous DNA without human intervention, we tested natural competence of the 11 environmental strains for the acquisition of the ARGCs *aadB* and *bla*_VIM-2_ at the native *attI1* site (see Methods). *Pseudomonas* sp. 1SL5, both *Acinetobacter* spp., and *Pantoea dispersa* 10FZSS14 isolates showed natural transformation for insertion of the *aadB* gene cassette, while *E*. *coli* 4IgSN1 also showed natural transformation for insertion of the *bla*_VIM-2_ gene cassette (Fig. 2c). Unexpectedly, this assay showed a high frequency (46%) and diversity of genera with natural competence for the insertion of wild-type ARGCs, which were against the prediction of the most accepted hypothesis. In a previous study, native chromosomal integrons from *P. stutzeri* strains 17587 *and* 17641 have been shown to acquire synthetic gene cassettes^39^ and environmental *A*. *baylyi* ADP1 has been shown to acquire complete genetic platforms of class 1 integrons^38^, both via natural transformation. Considering the large number of strains that possess not only *intI1* but also chromosomal integrons that have been estimated at more than 17% of the sequenced genomes through 2010^12^, these findings propose a scenario of a bacterial world opened up to a wide range of successful exchanges without human intervention. In agreement with this point of view, the evolutionary and ecological relevance of the processes of natural transformation and capture of ARGC by wild-type integrons, remains open to further investigation.

### Insights into the ARGCs molecular transfer

In order to determine the succession of molecular steps involved in the acquisition of ARGCs by *intI1*-positive environmental strains, we first tested for the presence of the donor paadB or pVIM2 plasmid in each environmental culture after 24 hrs of artificial and natural transformation of the plasmid (Fig. 2c). Maintenance of paadB and pVIM2 for more than 40 generations was identified in three independent experiments by PCR and sequencing of amplicons in both *Acinetobacter* spp., *E*. *coli* 4IgSN1 and *P*. *dispersa* 10FZSS14 (Fig. 2c, Supplementary Table S2). However, ARGC insertion was not observed in all the evolved environmental strains. As seen in Fig. 2c and d, subsequent insertion of the respective ARGC was observed for *aadB* in *E*. *coli* 4IgSN1, *Acinetobacter* 1IgSLAM1, *Acinetobacter* 1IgSN3 and *P*. *dispersa* 10FZSS14, while for *bla*_VIM-2_ it was found only inserted in the native *attI1* in *E*. *coli* 4IgSN1. Secondly, we performed RT-PCR in three independent experiments (at room temperature) to evaluate the expression of *intI1* in each environmental strain under four conditions: without the plasmid paadB, after artificial transformation with paadB, after artificial transformation process without paadB, and after natural transformation with paadB. Whereas only the *P*. *dispersa* 10FZSS14 isolate expressed the *intI1* gene without previous stresses, transcription was observed in some of the remaining environmental strains after transformation or exposure to DNA, which probably acted as stressors (Table 1). Lastly, to detect phenotypic changes of the environmental strains harbouring *aadB* or *bla*_VIM-2_ gene cassette in the native *attI1* site, we performed the selection after paadB and pVIM2 transformation with several concentrations of gentamicin and meropenem, and also exposed them to gradual sub-lethal concentrations of these antibiotics (Supplementary Table S1). Evolved *E*. *coli* 4IgSN1 strains have increased their MIC values of gentamicin and meropenem, since *E*. *coli* 4IgSN1::*aadB* and *E*. *coli* 4IgSN1::*bla*_*VIM*-*2*_ were selected with 16 μg/ml of gentamicin, and with 2 μg/ml of meropenem invaded by either *aadB*, or by *bla*_VIM-2_ gene cassette, respectively. Although no change in the susceptibility profile of the other evolved environmental strains was detected, the pending inquiry is to what extent sub-lethal concentrations of antibiotics may play an important role for the selection and maintenance of isolates harboring ARGCs along time in the open environment.

These results show an experimental setting in which a transient passage of plasmids in several environmental strains is enough not only to activate native *intI1* transcription that it is tightly regulated by LexA in several genomes^28, 47^ but also to achieve the delivery of the ARGC in the native *attI1* site^48^ (Fig. 2c). Expression of resistance followed by the insertion of ARGCs was not a common event in our environmental isolates, except in the case of *E*. *coli* 4IgSN1 (Supplementary Table S1), marking the relevance of antimicrobial resistance genomic pool as a “silent” reservoir of the open environment, even in habitats without human activity.

### No bacterial cost of ARGCs

**Figure 3:**
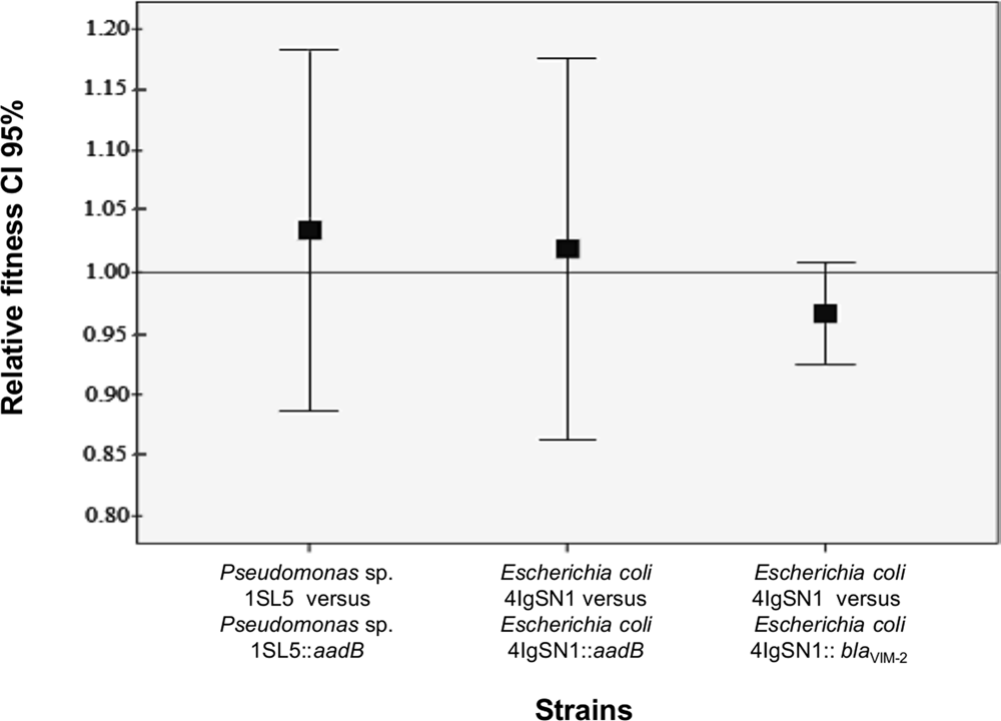
Pair-wise mixed culture competition experiments. Competence assays of environmental strains *Pseudomonas* sp. 1SL5 and *E*. *coli* 4IgSN1, against the corresponding ARGC (*aadB* or *bla*_VIM-2_) inserted at the native *attI1* site containing strain were conducted in order to determine the relative fitness. Error bars indicate 95% confidence intervals. By definition, a relative fitness (w) of 1.00 indicates no difference in relative fitness. Relative fitness values: *Pseudomonas* sp. 1SL5::*aadB* W = 1.03 (0.88–1.18), *E*. *coli* 4IgSN1::*aadB* W = 1.02 (0.86–1.17), *E*. *coli* 4IgSN1::*bla*_VIM-2_ W = 0.97 (0.92–1.01). CI is confidence interval.

We investigated whether there was biological cost for the acquisition of ARGCs on host environmental strains. The relative fitness (*w*) of the ancestor was by default set to 1.0. Mixed culture competition experiments were conducted with (i) *Pseudomonas* sp. 1SL5 competing with *Pseudomonas* sp. 1SL5::*aadB*, (ii) *E*. *coli* 4IgSN1 competing with *E*. *coli* 4IgSN1::*aadB*, and (iii) *E*. *coli* 4IgSN1 competing with *E*. *coli* 4IgSN1::*bla*_VIM-2_ (see Methods). Newly acquired ARGCs in *Pseudomonas* sp. 1SL5 and *E*. *coli* 4IgSN1 resulted in *w* values of 1.03, 1.02 and 0.97, respectively. These *w* values are close to 1.0, suggesting that the acquisition of ARGC has no detrimental cost for the environmental host (Fig. 3).

### Introducing the bidirectional model

Our study aimed to test the assumptions of a source-sink model which is the dominant paradigm of the flow of antimicrobial resistance genes between the hospital and the open environment habitats (Fig. 1).

However, our results did not support this model. Therefore, we propose an alternative model system based on a bidirectional flow of antibiotic resistance genes between the clinic and the open environment (Fig. 1). This model assumes that the environment contains (i) stable environmental populations of bacteria harbouring ARGCs (*w* ≥ 1), so the existence of putative resistant strains is not dependent on a steady flow from hospitals, and (ii) frequent lateral ARGC transfer processes mediated by active native IntI1s between bacterial species/genera in several environmental substrates, which may create new positive antimicrobial resistant strains. Two predictions of this bidirectional model are: (i) the open environment may function as a source of antibiotic resistance determinants, even when the contamination from the clinic is removed, and (ii) resistant strains that differ from those which colonize humans can disseminate from the open environment to the clinic.

### Local environmental surveillance to tackle ‘superbugs’

Many infection control policies have been suggested and some have been implemented in order to control and minimize the global threat of antimicrobial resistance^4, 49, 50^. Unfortunately, these efforts have not produced the expected results (http://www.cdc.gov/drugresistance/pdf/ar-threats-2013-508.pdf). Here, we found preliminary evidence of an active lateral ARGC transfer for different species from different microhabitats within natural and semi-natural environments. These results provide support for an eco-genetic model system that opens the possibility of a stable and systematic reintroduction of microbial resistance from the environment to the clinics, at least in the range of conditions that were investigated in our study. An essential point on which to focus for upcoming studies that has not been addressed to experimental level yet, is the study of the vectors involved in transferring the ARGCs-positive strains from the open environment to the hospital. Recently, the need for health organisations in each country has been stressed, which should be established with indigenous policies in order to control the huge rise of antimicrobial resistance^51^. If our results are confirmed and expanded to other genes and mobile genetic elements, in strains from other regions and other bacterial communities, promoting a sanitary program which includes an accurate surveillance of local epidemiology of determinants of resistance will be unavoidable.  This study should include both antimicrobial resistance genes and the genetic elements of the mobilome involved in the antimicrobial dissemination in the open environment at each geographical region in order to undertake local sanitary interventions. This sanitary program must cover antimicrobial resistance surveillance in animals, plants, and different substrates of the open environment.

Our results also offer experimental evidence of previous calls for attention from several specialists in the field in order to minimize the potential transference of resistance genes to the environment^5, 24–27, 52–56^, which points out many regulations such as: (1) eliminating the use of antibiotics as growth promoters, (2) reducing the use of antibiotics in animals and plants for therapeutic applications, (3) eliminating the use of disinfectants based on antibiotics outside the clinic, and (4) regulating the treatment of water and waste in a manner that minimizes the discharge of antimicrobial resistance genes and/or antibiotics to the environment, particularly from hospitals and industries. In this scenario, the rational use of antibiotics not only for humans but also for animals and agriculture with local policies should be mandatory. From an ecological perspective, a new paradigm of the interactions between humans and nature could be the key to solving the rapid and global spread of antimicrobial resistance determinants.

## Concluding remarks

Since there is a huge reservoir of gene cassettes in environmental bacteria^57^, our experimental data concerning the functionality of native IntI1s depicts the bacterial world as an interacting net of putative antimicrobial resistance mechanisms, with the capacity of some strains to maintain novel ARGCs after their acquisition without biological cost. In those regions where there has been a high incidence of nosocomial diseases and low sanitary control of personnel and waste, mechanisms linked to antibiotic resistance in the open environment can persist even after having achieved the disappearance of the resistant strains in the nosocomial niche. The bidirectional model system can also explain why the complete eradication of antimicrobial drugs in a nosocomial or veterinary habitat may not be sufficient to remove ARGCs in bacterial clinical populations, and can also help to enlighten the molecular processes involved in the emergence of different resistance genes in the clinic. Even more importantly, according to the molecular basis of this eco-genetic model system, new, unexpected lineages or species causing diseases that are resistant to antibiotics emerge.

## Methods

### Isolation sites

Environmental strains were isolated from two regions from Argentina separated by more than 4300 km: the south-eastern portion of Tierra del Fuego Island in Patagonia, and the north-eastern tip of the National Park of Iguazú, Misiones (Fig. 2). Tierra del Fuego Island lies within the Sub-Antarctic Deciduous Beech Forest, which is characterised by two species of southern beech, *Nothofagus pumilio* (Lenga) and *Nothofagus betuloides* (Guindo). Its climate belongs to the sub-polar oceanic type. Temperatures are cold all year round, with an average annual temperature of 5.7 °C and low annual temperature variations, ranging from −0.3 °C in July to 9.4 °C in January. The Iguazú National Park covers part of an ecoregion of the Paranaense Forest, also called the Upper Parana Atlantic Forest. The climate is tropical with dry season. Average temperatures range from 24 °C in summer to 14 degrees in winter. Its lush jungle is rich in lianas, epiphytes and ferns. Within these regions, 4 sample sites for Tierra del Fuego Island and 2 sample sites for Iguazú National Park were selected based on different levels of urbanization: low, medium and high. These levels were established following a quantitative procedure^22^.

### Sampling techniques, bacterial identification and detection of *intI1*-positive isolates

The environmental strains from this study were collected during two campaigns. Environmental samples from Tierra del Fuego Island were collected in February 2006 as previously described^22^. Faecal samples from foxes were taken also in this campaign. Environmental samples from the National Park of Iguazú were collected in February 2011. The methodology for the collection of samples was also the same. Shallow freshwater sediment and soil samples from the shore were plated on nutritive agar medium (Britania, Argentina) without antibiotics. The plates were incubated at room temperature for 1 day, after which all individual colonies from each site and from each plate were plated again in nutritive agar and incubated at room temperature for 1 day. Then, each colony was selected and identified using standard biochemical tests, microbiological test strips (API20NE-Biomerieux, France) and sequencing of the 16S rRNA gene using universal primers^58^. Concerning the sample technique of wild foxes´ faeces in Tierra del Fuego Island, they were observed and followed with binoculars until they had defecated. Then, the sample was taken with sterile swab and cultivated immediately at 37 °C for one day. A total of 4 faeces from 4 wild foxes had been sampled, from which we selected 25 colonies. Total DNA from 100 environmental samples from Tierra del Fuego Island from a previous study from our laboratory^22^ and 80 isolates from Iguazú National Park was extracted as previously described^40^ and Polymerase Chain Reactions (PCR) were carried out in 50 μl volumes for identification of the *intI1* gene. Each reaction contained 10 ng of DNA, 1X PCR buffer (Promega, USA), 0.2 mM of dNTPs mix (Genbiotech, Argentina), 0.4 μM of each primer (Genbiotech, Argentina), the necessary amount of sterile distilled water and 0.25 U *Taq* DNA polymerase (Promega, USA). The primers intI1F and intI1R (Supplementary Table S2) were used for amplification of the *intI1* gene, which produced a fragment of 925-bp length; also the primers *intI1 AvrII PstI* F and intI1RTR were used in order to obtain the complete sequence of the *intI1* gene of each one of the 11 environmental strains (Supplementary Tables S1 and S2). The PCR products were sequenced after purification using the Wizard SV Gel and PCR clean-up System kit according to the manufacturer’s protocol (Promega, USA); sequencing was performed on both DNA strands using ABIPrism 3100 BioAnalyzer equipment (Applied Biosystems, USA). The nucleotide sequences were analyzed using Blast V2.0 software (http://www.ncbi.nlm.nih.gov/BLAST/). From a total of 180 environmental isolates, 18 harboured the complete *intI1* gene. Eleven *intI1*-positive isolates, some of them from a previous study^22^, were selected for further studies: *Aeromonas media* 1AC2^22^, *Vibrio* sp. 1AC4^22^, *Pseudomonas* sp. 1SL5^22^, *Pseudomonas* sp. 7AN1^22^, *Aranicola* sp. 9AL34^22^, *Enterobacter* sp. 10AL1^22^, *Acinetobacter* sp. 1IgSLAM1, *Enterobacter* sp. 1IgSLAM2, *Acinetobacter* sp. 1IgSN3 and *Escherichia coli* 4IgSN1. One out of the 25 colonies isolated from foxes´ faecal samples was *intI1*-positive corresponding to *Pantoea dispersa* 10FZSS14 isolate, which was chosen for further studies. Before further studies, the presence of the *aadB* and *bla*_VIM-2_ gene cassettes had been investigated in each one of the 11 *intI1*-positive environmental strains by PCR with specific primers (Supplementary Table S2), rendering negative results (Data not shown).

### "﻿Clinical" or "﻿environmental" alleles of the *intI1* gene

The *intI1* genes can be an “environmental” or a “clinical” allele as defined by Gillings *et al*., suggesting a putative source from natural or clinical communities, respectively^9^. The same criterion was used in this article for *attI1* recombination sites (Supplementary Figure S1).

### Phylogenetic analysis of *intI1* alleles found in the environment and in the clinic

The phylogenetic tree was constructed using the Maximum Likelihood algorithm implemented in MEGA v6.0. The chosen model was T92 with uniform substitution rates among sites with 100 bootstraps.

### Plasmids used

Plasmid pMI1-1 is a derivative of pLQ369^59^ with a pMAL backbone (Amp^R^, New England Biolabs) containing *intI1* gene under the control of P_lac_ promoter and an *attI1* site cloned as the recombination target site for insertion of gene cassettes. It was constructed as follows: (i) the pCR2.1-TOPO polylinker with the *nde*I restriction site added, was amplified by using the M13F *nde*I and M13R *nde*I primers (Supplementary Table S2), and cloned into pCR2.1-TOPO using TOPO-TA cloning kit (Amp^R^ Kan^R^, Invitrogen) according to manufacturer's indications, creating pCpoly AS; (ii) the pCpoly AS insert was cloned into pLQ369, both digested with *nde*I, yielding pLQ3poly; (iii) the 65 bp *attI1*-carrying fragment was amplified from *Serratia marcescens* strain SCH909 by using the *attI1 Avr*II F and *attI1* (*qacE*) *Not*I R primers (Supplementary Table S2), and cloned into pCR2.1-TOPO, creating pC1 AS; and (iv) the *not*I fragment from pC1 AS, carrying *attI1*, was cloned into pLQ3poly, yielding pMI1-1. ARGC donor plasmids in recombination assays, paadB and pVIM2, carrying *aadB* or *bla*_VIM-2_ ARGC, respectively, were constructed using TOPO-TA cloning kit (Invitrogen) according to manufacturer's indications. PCR products containing each ARGC were ligated to pCR2.1-TOPO vector as indicated by Invitrogen’s protocol: (i) paadB plasmid, *Serratia marcescens* strain Sm404 has a class 1 integron with the *aadB* ARGC at the variable region. Amplicon containing *aadB* ARGC was generated using primers attI1F and 3′CS (Supplementary Table S2) spanning 65-bp of the *attI1* target site, the complete *aadB* ARGC and 100 pb of 3′CS; (i) pVIM2 plasmid, PCR amplicon carrying *bla*_VIM-2_ cassette was obtained from *Pseudomonas aeruginosa* strain PAE981. This strain possesses a class 1 integron with the array *bla*_VIM-2/_ *aac*(*6*′)-*Ib*/*aacA7* in the variable region (Data not shown). Expected amplicon was recovered with primers attI1F and aac6′R (Supplementary Table S2). Resulting PCR amplicon contained 65 bp of the *attI1* site, the complete *bla*_VIM-2_ ARGC and 505 bp of the 5′ region of *aac*(*6*′)-*Ib* ARGC.

### Transformation assays

Chemical transformation was performed for the 11 environmental strains^40^ with paadB and pVIM2 plasmids in independent assays. Briefly, each environmental strain (Supplementary Table S1) was made competent with CaCl_2_ and aliquoted in 50 µl in 0.6 PCR tubes for each transformation. This aliquot was incubated 30 minutes on ice in the presence of each plasmid (paadB or pVIM2). After this incubation, the tubes were exposed to a 90-second heat-shock at 42 °C to be transformed. Then, 200 µl of LB medium was added and incubated at room temperature for 2 hours in the case of environmental strains or 1 hour at 37 °C for *E*. *coli* TOP10 pMI1-1. Transformation process was performed without antibiotic selection in order to simulate natural, non-clinical conditions. For environmental strains and also for assays without selection of *E*. *coli* TOP10 pMI1-1, 50 μl of transformed bacteria were plated in LB agar, incubated overnight at room temperature in the case of environmental strains and at 37 °C for *E*. *coli* TOP10 pMI1-1. Some colonies of each environmental strain were used to inoculate a 5-ml LB broth. Besides, one assay using *E*. *coli* TOP10 previously transformed with pMI1-1 as recipient was performed with antibiotic selection; 50 μl of transformed bacteria were plated in LB agar supplemented with kanamycin 25 µg/ml, incubated overnight and some colonies were used to inoculate a 5-ml LB broth. Then, total DNA extraction^40^ was performed with the 24-hours 5-ml LB broth in order to achieve molecular studies (see below).

### Confirmation of the insertion of the ARGC within native *attI1* sites

Recombination mediated by native IntI1 was evaluated by PCR and sequencing using the primers insF and aadB5′R or vim2-5′R (Supplementary Table S2), according to the tested ARGC (*aadB* or *bla*_VIM-2_, respectively). Five μl of the total DNA extraction of each environmental strain performed with the 24-hours 5-ml LB broth post-transformation with paadB or pVIM2 (see above) was used to confirm ARGC insertion for PCR assay. The PCR products were purified using the Wizard SV Gel and PCR clean-up System kit according to the manufacturer’s directions (Promega, USA); the sequencing was performed on both DNA strands using ABIPrism 3100 BioAnalyzer equipment (Applied Biosystems, USA). The nucleotide sequences were analyzed using Blast V2.0 software (http://www.ncbi.nlm.nih.gov/BLAST/).

### Detection of paadB and pVIM2 in environmental strains

Plasmid presence was analyzed using primers pairs blaF-blaR (Supplementary Table S2). As described above, 5 ul of the total DNA extraction performed with the 24-hours 5-ml LB broth post-transformation (see above) was used to confirm plasmid acquisition by environmental transformed strains. PCR products were purified and sequenced as indicated before. The PCR products were purified using the Wizard SV Gel and PCR clean-up System kit according to the manufacturer's instructions (Promega, USA); the sequencing was performed on both DNA strands using ABIPrism 3100 BioAnalyzer equipment (Applied Biosystems, USA). The nucleotide sequences were analyzed using Blast V2.0 software (http://www.ncbi.nlm.nih.gov/BLAST/).

### Determination of antimicrobial resistance acquisition of environmental strains

Antibiotic resistance acquisition following ARGC insertion was determined by plating 50 μl of 24-hours 5-ml LB broth in LB agar supplemented with gentamicin or meropenem depending on the case (*aadB* or *bla*_VIM-2_ insertion). The antibiotic concentration used was one and two dilutions greater than the CIM of each isolate (Supplementary Table S1).

### Insertion frequency measured by Real Time PCR

ARGC insertion frequency was estimated by the ratio of the insertion number and the total *intI1* number. In order to identify insertions, the primers insF and aadB5′R or vim2-5′R were used according to the tested ARGC (as indicated before); *intI1* number was detected using intI1RTF and intI1RTR primers (Supplementary Table S2).

The insertion number and total *intI1* genes were estimated by standard curves for each gene plotting DNA concentration (copy number µl^−1^) against *C*_t_ value. Known copy number standards consisting of 2 × 10^9^ amplicon copies μL^−1^ and four ten-fold serial dilutions for each amplicon were prepared. Copy number μL^−1^ was calculated making use of the respective molar mass. These standard curves were included in every Real Time PCR run. Foremost, insertion and *intI1* fragments were obtained by conventional PCR from positive controls. The *intI1* gene and *aadB* insertion amplicons were taken from a *Serratia marcescens* strain carrying a class 1 integron with an *aadB* cassette in first position of its variable region. On the other hand, *bla*_VIM-2_ cassette insertion amplicon was obtained from a *Pseudomonas aeruginosa* PAE981 class 1 integron positive strain holding *bla*_VIM-2_ ARGC in first place in the variable region. NucleoSpin Gel and PCR Clean-up kit (Macherey-Nagel) was used for amplicon purification and quantification was assessed by spectrophotometry. With the *C*_t_ values obtained from both standard groups, a regression analysis was performed to generate the line equation and, therefore, estimate the copy number in every recombination assay.

Real Time PCR was carried out in a Rotor Gene 6000 (Corbett Life Science) using 0.2-μL PCR tubes. Each reaction contained 1X HOT FIREPol EvaGreen qPCR Mix Plus (No ROX), 0.1 μM each primer, and 2 μL of DNA, made up to a final volume of 20 μL with bidistilled water. The PCR protocol consisted of an initial denaturation of 94 °C for 3 min, followed by 40 cycles of 94 °C for 15 s, annealing (56 °C for cassette insertion and 52 °C for *intI1* gene) of 30 s and a 72 °C-extension for 20 s when the acquired fluorescence was measured. Finally, the melt curve was performed, in which the specificity of all reactions was checked. The threshold was manually adjusted to 0.05 and the first five cycles were eliminated to discard incorrect *C*_t_ values.

### Testing Natural Transformation

Natural transformation was tested in all the environmental strains (Supplementary Table S1). Then, 50 µl of a 24-hour LB broth containing each isolate was incubated for 2 hours with 50 µl containing 400 ng of paadB or pVIM2 plasmids. After this, 100 µl of culture was used to inoculate a 3-ml LB broth without antibiotics for 24 hours. Detection of plasmid acquisition was executed by plating an aliquot of each assay in LB agar in an appropriate dilution to determine the presence of the plasmid by PCR with the respective primers (Supplementary Table S2). ARGC insertion following natural transformation was detected from total DNA extraction of the final culture by PCR and sequencing using the appropriate primer pair, as described above.

### Maintenance of paadB and pVIM2 plasmids in environmental strains

The eleven environmental strains (Supplementary Table S1) were analyzed for the maintenance of paadB or pVIM2. For this assay, each environmental strain was cultured at room temperature overnight into 5 ml L broth. The culture was then diluted 10^−6^-fold and cultured again at room temperature overnight. This cycle was repeated an appropriate number of times, with each time being 40 generations. The growing colonies were then tested for presence of plasmid by detecting by PCR with blaF/blaR primers (Supplementary Table S2). Experiments were performed three times, and 30 colonies per generation were assayed.

### RT-PCR

RNA was extracted using the Trizol method (Invitrogen, CA) finishing with a RQ1 RNAse free DNAse (Promega) treatment. The cDNA was made using ImProm-II RT (Promega) and the PCR was performed as indicated before, adding 1 µl of cDNA as template. *intI1* transcription was evaluated using primers intI1RTF and intI1RTR (Supplementary Table S2) in environmental strains and *Escherichia coli* TOP10 pMI1-1 in wild type state, post-chemical transformation, post-chemical transformation process without any plasmid and post-natural transformation.

### Fitness assays

In order to evaluate the biological cost of the ARGC, evolved *aadB* or *bla*_VIM-2_ positive environmental isolates were used (*Pseudomonas* sp. 1SL5::*aadB*, *E*. *coli* 4IgSN1::*aadB* and *E*. *coli* 4IgSN1::*bla*_VIM-2_, respectively). To select *Pseudomonas* sp. 1SL5::*aadB* which did not express gentamicin resistance (Supplementary Table S1), 30 colonies from one plate without antibiotic were picked for colony PCR with primers insF and aadB5′R (Supplementary Table S2) in order to detect the positive evolved strain. In contrast, *E*. *coli* 4IgSN1::*aadB* and *E*. *coli* 4IgSN1::*bla*_VIM-2_ were selected on LB agar with gentamicin or meropenem, respectively, after the corresponding transformation with paadB or pVIM2.

To perform fitness assays^60^, some modifications were made for *Pseudomonas* sp. 1SL5 competing with *Pseudomonas* sp. 1SL5::*aadB*. This strain did not show resistance to gentamicin after *aadB* gene cassette acquisition, so the amount of competitor was determined by colony PCR using insF and aadB5′R primers (Supplementary Table S2). In this case, each competitor was identified by the presence or absence of amplicons, *Pseudomonas* sp. 1SL5::*aadB* or wild *Pseudomonas* sp. 1SL5, respectively (primers insF and aadB5′R; Supplementary Table S2). The Malthusian parameter^61^ was determined using the equation M = ln (N_1_/N_0_)/1 day. The relative fitness of the competitors is the ratio between M from the strain that acquired the gene cassette and that of the wild type strain. Fitness determination was performed ten times for each pair of competitors. The integrity of each ARGC, *aadB* or *bla*_VIM-2_ gene cassettes, was checked by PCR and sequencing with specific primers targeting the *attI1* site and the respective ARGC described in Supplementary Table S2 in 30 out of 100 colonies in each independent experiment of *Pseudomonas* sp. 1SL5::*aadB*, *E*. *coli* 4IgSN1::*aadB* and *E*. *coli* 4IgSN1::*bla*_VIM-2_ strains, respectively, after the fitness experiments.

### Susceptibility testing

Minimal inhibitory concentration of gentamicin and meropenem was determined for all isolates, plating them in Muller Hinton agar, as indicated by CLSI with different antibiotic concentration ranging from 0.125 to 64 µg/ml (Supplementary Table S1). The inoculums used were the standard ones (0.5 McFarland turbidity standards).

## Electronic supplementary material

Supplementary Information
