# Supplementary material for: Lateral Antimicrobial Resistance Genetic Transfer is active in the open environment
Source: Sci Rep. 2017 Mar 31;7:513. doi: 10.1038/s41598-017-00600-2 (PMC5428826; doi:10.1038/s41598-017-00600-2)
Supplement: Supplementary file 1 — Supplementary Information [file 41598_2017_600_MOESM1_ESM.pdf]

Lateral Antimicrobial Resistance Genetic Transfer is active in the open environment

Luciana S. Chamosa[1], Verónica E. Álvarez[1], Maximiliano Nardelli[1], María Paula Quiroga[1], Marcelo H. Cassini[2, 3] and Daniela Centrón[1*].

[1]Instituto de Microbiología y Parasitología Médica, Facultad de Medicina, Universidad de Buenos Aires-Consejo Nacional de Investigaciones Científicas y Tecnológicas (IMPaM, UBA-CONICET), Ciudad Autónoma de Buenos Aires, Argentina;

[2]Grupo GEMA, Departamento de Ciencias Básicas, Universidad Nacional de Luján, Luján, Buenos Aires, Argentina

[3]Laboratorio de Biología del Comportamiento, IBYME, Ciudad Autónoma de Buenos Aires, Argentina.

Supplementary Table S1. Characteristics of the *intI1*-positive environmental strains from this study.

Isolate	Genotypic Characterization[a]	Accession Number	MIC	
			Gentamicin	Meropenem
Pseudomonas sp. 1SL5	99.8% *Pseudomonas poae* (AN HQ898911)	JN870908	20 μg/ml	16 μg/ml
Pseudomonas sp. 7AN1	99.86% *Pseudomonas fragi* (AN JX867753)	JN870912	0.5 μg/ml	1 μg/ml
Aeromonas media 1AC2	100% *Aeromonas media* (AN JX845724)	JN870911	0.5 μg/ml	32 μg/ml
Vibrio sp. 1AC4	99.8% *Vibrio metschnikovii* (AN JX409930)	JN870903	0.25 μg/ml	0.5 μg/ml
Aranicola sp. 9AL34	99.5% *Aranicola* sp. Ct2 (AN GU391491)	JN870904	3 μg/ml	0.5 μg/ml
Enterobacter sp. 10AL1	98.7% *Enterobacter ludwigii* (AN JX215328)	JN870907	12 μg/ml	0.5 μg/ml
Enterobacter sp. 1IgSLAM2	99.48% *Enterobacter asburiae* (AN EU543690)	KJ701244	1 μg/ml	4 μg/ml
Escherichia coli 4IgSN1	100% *Escherichia coli* (AN JN129478)	KJ701246	8 μg/ml	0.2 μg/ml
Pantoea dispersa 10FZSS14	100% *Pantoea dispersa* (AN JQ659939)	KJ701247	0.5 μg/ml	64 μg/ml
Acinetobacter sp. 1IgSN3	99.23% *Acinetobacter baylyi* (AN JQ229812)	KJ701245	16 μg/ml	1 μg/ml
Acinetobacter sp. 1IgSLAM1	99.0% *A. calcoaceticus* (AN HE610784)	KJ701243	16 μg/ml	1 μg/ml
Pseudomonas sp. 1SL5::*aadB*	*aadB* cassette inserted in the native *attI1* site	NA	20 μg/ml	ND
Escherichia coli 4IgSN1::*aadB*	*aadB* cassette inserted in the native *attI1* site	NA	32 μg/ml	ND
Escherichia coli 4IgSN1::bla_{VIM-2}	bla_{VIM-2} cassette inserted in native *attI1* site	NA	ND	4 μg/ml

The 11 *intI1*-positive environmental strains were characterized as sp., unless a 100% homology in the 16S rDNA was detected. All 11 *intI1*-positive environmental isolates were negative for *aadB* and bla_{VIM-2} genes (Supplementary Table S2). *Pseudomonas* sp. 1SL5, *Pseudomonas* sp. 7AN1, *Aeromonas media* 1AC2, *Vibrio* sp. 1AC4, *Aranicola* sp. 9AL34 and *Enterobacter* sp. 10AL1 were recovered and analyzed in a previous study conducted by our group[22]. *Escherichia coli* 4IgSN1::*aadB* and *Escherichia coli* 4IgSN1::bla_{VIM-2} were obtained by selection on plate supplemented with gentamicin or meropenem, respectively. *Pseudomonas* sp. 1SL5::*aadB* was isolated by colony PCR screening because it did not express gentamicin resistance (see Methods). NA: not applicable. ND: not determined. [a] Identity percentage with the most related organism by 16S rDNA sequence with the corresponding Accession Number indicated in parentheses. [b] Novel int*I1* alleles were defined as "environmental" whereas the *intI1* genes that showed identity with previous alleles from the clinic were defined as "clinical" alleles[23].

Supplementary Table S2. Primers used in this study.

Target	Primer pair	Sequence 5'- 3'	AN and position[a]	Reference
16S rDNA	Rp2	ACGGCTACCTTGTTACGACTT	Accession Number HM107075: 1 - 1511	[58]
	Fd2	AGAGTTTGATCATGGCTCAG		[58]
intI1	intI1R	TTCGAATGTCGTAACCGC	Accession Number AJ871915: 265 - 1190	[22]
	intI1F	CGAGGCATAGACTGTAC		[22]
	intI1RTF	GCGTGTAAATCATCGTCGTAG	Accession Number AJ871915: 211 - 396	This work
	intI1RTR	TGTATGACCAGACCTTTCAGC		This work
	intI1 AvrII PstI F	CCTAGGCTGCAGTCCATCAGGCAACGACGGGC	Accession Number AJ871915: 4 – 23	This work
Cloning pCR2.1 TOPO polylinker	M13F *nde*I	CATATGGTAAAACGACGGCCAG	pCR2.1-TOPO vector (Invitrogen): 205 – 406	This work
	M13R *nde*I	CATATGCAGGAAACAGCTATGAC		This work
Cloning *attI1* site	*attI1 Avr*II F	ATAAACCTAGGCGTTACGCCGTGGGTCG	Accession Number AF453998: 905 – 987	This work
	attI1 (*qacE*) *Not*I R	AATATAAAGCGGCCGCTGCATCTAACTTTGTTTTAG		This work
aadB	aadBF	GTAACACGCAAGCACGATGA	Accession Number AJ871915: 1407 – 1757	This work
	aadBR	GCCTGTAGGACTCTATGTGC		This work
bla$_{VIM-2}$	VIM2F	TGGGCCATTCAGCCAGATC	Accession Number JX120362: 1606 – 2115	This work
	VIM2R	ATGGTGTTTGGTAGCATATC		This work
Cloning *aadB* cassette	attI1F	GCGTTACGCCGTGGGTCG	Accession Number JX494728: 76 – 801	This work
	3'CS	AAGCAGACTTGACCTGA		[62]
Cloning *bla*$_{VIM-2}$ cassette	attI1F	GCGTTACGCCGTGGGTCG	Accession Number AY029772: 444 – 1261	This work
	aac6'R	GTGTTCGCTCGAATGCC		This work
Cassette donor plasmid	M13F	GTAAAACGACGGCCAG	pCR2.1-TOPO vector (Invitrogen): 205 – 406	Invitrogen
	M13R	CAGGAAACAGCTATGAC		Invitrogen
	blaF	GTGCTCATCATTGGAAAACG	pCR2.1-TOPO vector (Invitrogen): 2227 – 2336	This work
	blaR	GATGCTGAAGATCAGTTGGG		This work
aadB insertion	insF	TTTGTACAGTCTATGCCTCG	Accession Number AJ871915: 1170 -1525	This work
	aadB5'R	AAGAATCCATAGTCCAACTCC		This work
bla$_{VIM-2}$ insertion	insF	TTTGTACAGTCTATGCCTCG	Accession Number JX120362: 1308-1514	This work
	vim2-5'R	CGCAATAGCCATGATAGACG		This work

Genetic characterization of the 11 *intI1*-positive environmental strains was performed amplifying by PCR and sequencing 16S rDNA with primers Rp2 and Fd2. The *intI1* detection was done by PCR with primers int1F and intI1R. The complete sequence of this gene (1014 pb) from each isolate was performed with two primer sets: intI1F-intI1R, and *intI1 AvrII PstI* F- intI1RTR. The primers

intI1RTF and intI1RTR were used for the quantification and transcription evaluation of this gene. The M13F *nde*I and M13R *nde*I primers were used for cloning the pCR2.1 TOPO polylinker (pCpoly AS). The *attI1 Avr*II F and *attI1* (*qacE*) *Not*I R primers were used for cloning the *attI1* site (pC1 AS). ARGCs were cloned using primers attI1F combined with 3'CS for *aadB* (paadB) or aac6'R for bla_{VIM-2} (pVIM2), respectively (see Methods). ARGC insertion analysis was identified with primers insF and aadB5'R (for *aadB* gene cassette) or vim2-5'R (for bla_{VIM-2} gene cassette). The blaF/blaR primers were used to evaluate plasmid maintenance. The M13F and M13R primers were used to detect ARGC excision. Combination of primers insF with aadB5'R or insF with vim2-5'R were used according to the tested ARGC for frequency studies by Real time PCR; the *intI1* number was detected using intI1RTF and intI1RTR primers by Real time PCR. Fitness analysis of *Pseudomonas* sp. 1SL5::*aadB* and *Pseudomonas* sp. 1SL5 competitors were identified by the presence or absence of amplicons, by using primers insF and aadB5'R. Primers aadBF and aadBR were utilized in *aadB* cassette transcription evaluation.

[a] Reference accession number AN and position of the fragment amplified.

References

22 Nardelli, M. *et al.* Class 1 integrons in environments with different degrees of urbanization. *PLoS One* **7,** e39223, doi:10.1371/journal.pone.0039223 (2012).

23 Gillings, M. R., Krishnan, S., Worden, P. J. & Hardwick, S. A. Recovery of diverse genes for class 1 integron-integrases from environmental DNA samples. *FEMS Microbiol. Lett.* **287,** 56-62, http://dx.doi.org/10.1111/j.1574-6968.2008.01291.x (2008).

58 Weisburg, W. G., Barns, S. M., Pelletier, D. A. & Lane, D. J. 16S ribosomal DNA amplification for phylogenetic study. *J. Bacteriol.* **173,** 697-703 (1991).

62 Hannecart-Pokorni, E. *et al.* Characterization of the 6'-N-aminoglycoside acetyltransferase gene *aac(6')-Im* [corrected] associated with a sulI-type integron. *Antimicrob. Agents Chemother.* **41,** 314-318 (1997).

```
"Clinical " allele          CGATGTTTGATGTTATGGAGCAGCAACGATGTTACGCAGCAGGGCAGTCGCCCTAAAACA 60
Acinetobacter sp. 1IgSN3    CGATGTTTGATGTTATGGAGCAGCAACGATGTTACGCAGCAGGGCAGTCGCCCTAAAACT 60
Aranicola sp. 9AL34         CGATGTTTGATGTTATGGAGCAGCCACGATGTTACGCAGCAGGGCAGTCGCCCTAAAACA 60
                            ************************.**********************************:

"Clinical " allele          AAGTT 65
Acinetobacter sp. 1IgSN3    AAGTT 65
Aranicola sp. 9AL34         AAGTT 65
                            *****
```

Supplementary Figure S1. Multiple alignment of *attI1* sites. *Acinetobacter* sp. 1IgSN3 and *Aranicola* sp. 9AL34 strains had two novel "environmental" *attI1* site variants with a single mutation respect of the "clinical" variant (DQ247972). The remaining 9 *intI1*-positive environmental strains possessed the "clinical" *attI1* site (DQ247972). The multiple alignment was performed using ClustalW2 online tool on the EMBL-EBI web site.
